# Staying in touch: how highly specialised moth pollinators track host plant phenology in unpredictable climates

**DOI:** 10.1186/s12862-021-01889-4

**Published:** 2021-08-24

**Authors:** Jonathan T. D. Finch, Sally A. Power, Justin A. Welbergen, James M. Cook

**Affiliations:** Hawkesbury Institute for the Environment, Western Sydney University, Richmond, NSW Australia

**Keywords:** Nursery pollination, *Epicephala*, *Breynia*, Diapause, Obligate pollination mutualism

## Abstract

**Background:**

For specialised pollinators, the synchrony of plant and pollinator life history is critical to the persistence of pollinator populations. This is even more critical in nursery pollination, where pollinators are obligately dependant on female host plant flowers for oviposition sites. *Epicephala* moths (Gracillariidae) form highly specialised nursery pollination mutualisms with Phyllanthaceae plants. Several hundred Phyllanthaceae are estimated to be exclusively pollinated by highly specific *Epicephala* moths, making these mutualisms an outstanding example of plant–insect coevolution. However, there have been no studies of how *Epicephala* moths synchronise their activity with host plant flowering or persist through periods when flowers are absent. Such knowledge is critical to understanding the ecology and evolutionary stability of these mutualisms. We surveyed multiple populations of both *Breynia oblongifolia* (Phyllanthaceae) and it’s *Epicephala* pollinators for over two years to determine their phenology and modelled the environmental factors that underpin their interactions.

**Results:**

The abundance of flowers and fruits was highly variable and strongly linked to local rainfall and photoperiod. Unlike male flowers and fruits, female flowers were present throughout the entire year, including winter. Fruit abundance was a significant predictor of adult *Epicephala* activity, suggesting that eggs or early instar larvae diapause within dormant female flowers and emerge as fruits mature. Searches of overwintering female flowers confirmed that many contained pollen and diapausing pollinators. We also observed diapause in *Epicephala* prior to pupation, finding that 12% (9/78) of larvae emerging from fruits in the autumn entered an extended diapause for 38–48 weeks. The remaining autumn emerging larvae pupated directly without diapause, suggesting a possible bet-hedging strategy.

**Conclusions:**

*Epicephala* appear to use diapause at multiple stages in their lifecycle to survive variable host plant phenology. Furthermore, moth abundance was predicted by the same environmental variables as male flowers, suggesting that moths track flowering through temperature. These adaptations may thereby mitigate against unpredictability in the timing of fruiting and flowering because of variable rainfall. It remains to be seen how widespread egg diapause and pre-pupal diapause may be within *Epicephala* moths, and, furthermore, to what degree these traits may have facilitated the evolution of these highly diverse mutualisms.

**Supplementary Information:**

The online version contains supplementary material available at 10.1186/s12862-021-01889-4.

## Background

In nature, resources are often ephemeral and unpredictable. How species use ephemeral resources, such as prey, fruits or flowers, is an important question in ecology [1–3]. Flowering can be influenced by a variety of environmental factors including temperature, rainfall and photoperiod [4–6]. The timing and intensity of some environmental events, however, can be highly variable among years, making the distribution and occurrence of flowering resources unpredictable. Pollinating insects that rely on a small number of flowering species, so-called specialists or oligotrophs, may be at greater risk of extinction due to a lack of available flowers [7].

Nursery pollination mutualisms are perhaps the most specialised interactions known to occur between plants and insect pollinators [8, 9]. In nursery pollination, insect pollinators transport pollen between the male and female flowers of a single host plant species. Along with pollen, female pollinators also deposit their eggs into female flowers. The ovules of the developing fruit then become the nursery and primary food source for the pollinator’s offspring. As pollinator larvae can only usually develop within the fruits of a single host plant, adult pollinators are obligately dependant on flowers to complete their life cycles.

Many forms of nursery pollination are currently known, the most widely studied being those occurring in figs [10], *Yucca* [11]*,* globeflowers [12] and some members of the Phyllanthaceae family [13]. The nursery pollination mutualisms occurring within the Phyllanthaceae or “leaf flowers” are the most recently discovered (~ 20 ya) of the major mutualisms [14], and it is now believed that up to 700 species of the genera *Breynia, Glochidion* and *Phyllanthus* are pollinated exclusively by *Epicephala* moths (Gracillariidae), also known as leaf flower moths [15–17]. The nursery mutualisms occurring between *Epicephala* moths and the leaf flowers are believed to have evolved once in Asia around 23 mya before rapidly diversifying and spreading across Asia, Oceania and the Neotropics [15, 16]. As such, these interactions are perhaps one of our most outstanding examples of plant–insect coevolution.

In nursery pollination, the synchrony of plant and pollinator life history is critical to the persistence of pollinator populations and the stability of the mutualism [18]. Within plants that use nursery pollination, there is a broad spectrum of flowering activity. Plants in the tropics can flower continuously or near continuously [18–20], whilst those in the sub-tropics and temperate regions may flower as little as once per year [21, 22]. Meanwhile, desert-dwelling *Yucca* species may not flower for several years at a time [11]. In many tropical fig species, individual trees flower asynchronously throughout the year, resulting in continuous year round flowering at the population level [18, 23–25]. Indeed, the continuous flowering of tropical fig trees is critical to prevent local fig wasp extinction, as a constant supply of syconia is required to maintain stable pollinator populations [18, 23, 26]. In temperate fig trees, where flowering only occurs during the spring and summer, fig wasps overwinter in “dormant” figs and emerge the following spring [22]. As such, an important question is how are pollinator populations maintained in leaf flower nursery mutualisms, where flowering often occurs in discrete episodes and not continuously throughout the year?

Populations of pollinators in nursery mutualisms have rarely been surveyed [18]. From the few available observations, it would seem that *Epicephala* abundance peaks following periods of host plant fruiting [19–21]. This makes intuitive sense, given that *Epicephala* larvae develop by feeding on growing fruits. However, moths that pollinate plants with discrete and seasonal flowering and fruiting times cannot rely on a continuous supply of receptive female flowers to maintain their population. It is therefore likely that they have evolved mechanisms to cope with large gaps in time between fruiting and flowering. Many moths, including at least one species of *Epicephala,* are known to undergo diapause at the egg or pre-pupal stages as a mechanism for bridging the time between fruiting and flowering [21, 27–29]. In the *Yucca*-yucca moth mutualisms, moths can remain in pre-pupal diapause for up to 4 years [11]. It seems likely, therefore, that *Epicephala* moths may use some form of diapause during these flowering-fruiting gaps.

If diapause does occur in *Epicephala* moths, then we should expect that it is induced and broken by the same environmental factors that influence flowering. This is because many species of Lepidoptera are known to be phenologically synchronised with their host plants via climate cues, such as temperature [30–34]. To date there have been no studies of how *Epicephala* moths synchronise their lifecycle with those of their host plants, or the environmental factors that influence these interactions.

We set out to determine the annual activity of *Epicephala* moth pollinators on their obligate host plant, *Breynia oblongifolia* [35, 36]. *Breynia oblongifolia* is generally regarded to flower and fruit throughout the austral spring, summer, and autumn (September to May), meaning that *Epicephala* moths are likely to experience a lack of available flowers during the winter months. However, it is unknown how these *Epicephala* moth populations persist through periods of time in which flowers are absent. We surveyed both pollinators and host plants for more than two years to determine their phenology. We hypothesized that because of the obligate dependence of *Epicephala* moths on *B. oblongifolia* fruits and flowers, moth abundance would closely track flowering phenology.

Rainfall in Australia can be highly variable between years [37–39]. As such, many species of Australian plant are known to fruit and flower in response to variation in rainfall and photoperiod [4, 5]. We modelled the environmental factors that drive plant and pollinator phenology, predicting that the abundance of adult moths and flowers would be best predicted by rainfall and photoperiod. Then, using our newly gained knowledge of moth and host plant phenology, we looked for evidence of diapause at both the egg and pre-pupal stages. In undertaking this study, we sought to understand how highly host-specific pollinators track host plant phenology and maintain their populations over time.

## Methods

### Study species

**Fig. 1:**
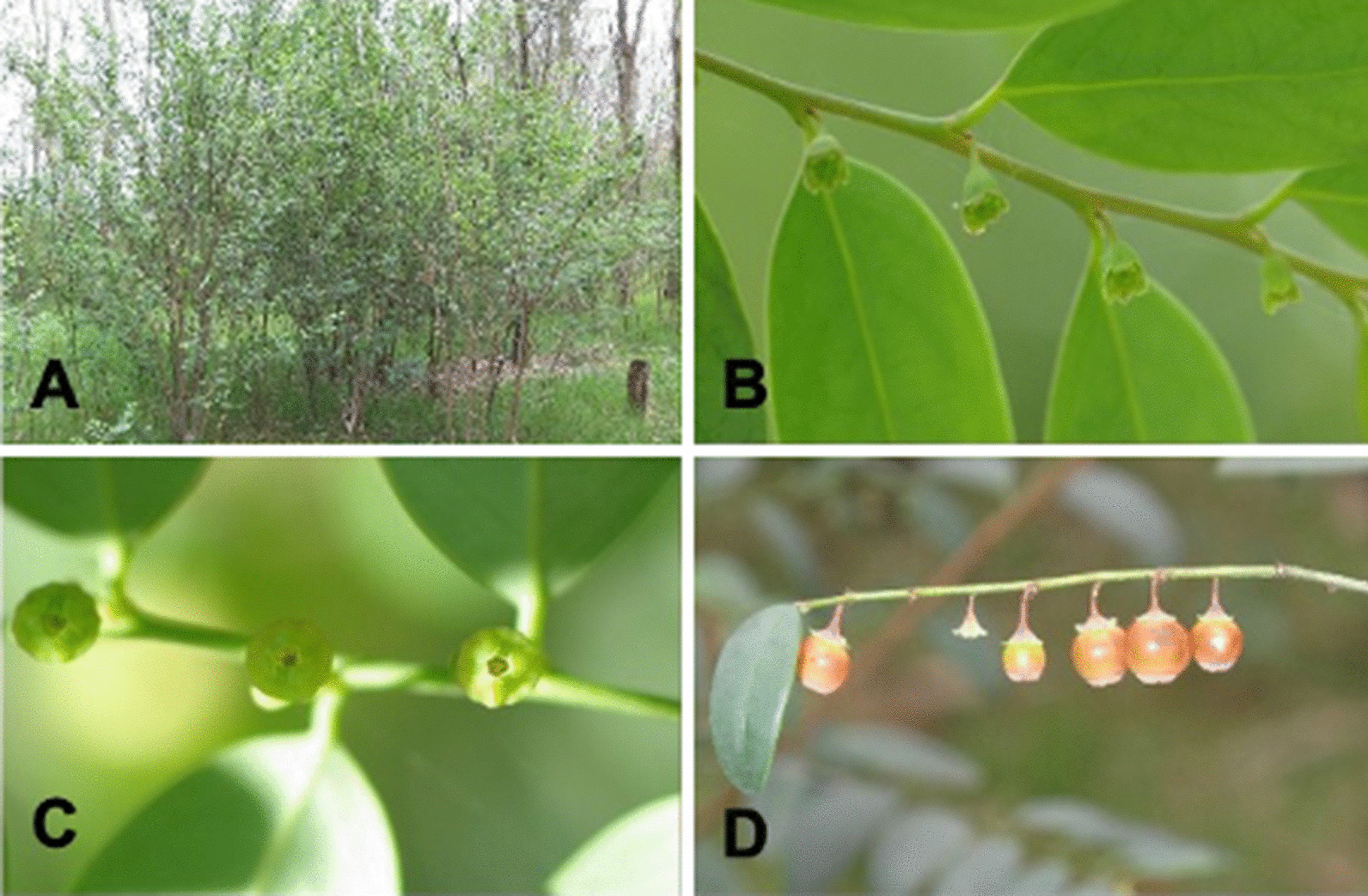
*Breynia oblongifolia*. Mature plants **A** female flowers, **B** female flowers, **C** male flowers, **D** mature and developing fruits. All photographs by JF

*Breynia oblongifolia* occurs along the east coast of Australia from southern New South Wales to northern Queensland [40]. Mature plants are generally 1–3 m tall and bear unisexual male and female flowers that emerge from the leaf axils (Fig. 1). In male flowers, the stamens are almost entirely enclosed by fused sepals, with only a narrow aperture at the apex allowing access to pollinators. *B. oblongifolia* is known to be pollinated by at least two closely related species of *Epicephala* moth [35, 36]. Many species of *Epicephala* have been described from Australia. However, the original species descriptions often lack information on genital morphology, which is now considered essential for their identification [41, 42]. As such, the taxonomic identity of the two *Epicephala* species present on *B. oblongifolia* is currently unknown and they are referred to as *Epicephala* sp. A and *Epicephala* sp. B. At the time of pollination, female moths oviposit eggs into female flowers. *Epicephala* larvae feed on the developing ovules with a single larva consuming around half the seeds in each fruit [35, 36]. *Epicephala* moths emerge from mature fruits as larvae and pupate on the foliage or leaf litter. However, lifecycle details of these highly specific pollinators are largely unknown.

*Breynia oblongifolia* is a non-protected plant species in New South Wales under the Biodiversity Conservation act of 2016. The identification and collection of all plant material was made by JF on land owned and managed by Western Sydney University*.* No specific collection permissions were required.

### Flowering and fruiting phenology

To determine the occurrence and environmental drivers of fruiting and flowering, we recorded the reproductive phenology of four populations of *B. oblongifolia* at two coastal and two inland sites in NSW, Australia: Richmond, Shellharbour, Shoal Bay and Millfield (Table 1). To ensure the independence of environmental effects between sites, we chose four populations that were at least 90 km apart. All four populations were situated in *Eucalyptus*-dominated woodlands, where *B. oblongifolia* occurs as a component of the understory and had at least 50 flowering plants. Populations at Richmond were surveyed every 2–4 weeks between September 2015 and April 2018. Other populations were surveyed at intervals of approximately 30 days between November 2016 and November 2017. At each site 20 plants were chosen by walking along a pre-determined transect line, either a road or footpath, and choosing the nearest plant > 1 m in height every 10 m. Four branches, approximately 30 cm in length, were selected on each plant and marked for monitoring by repeat surveys. On each subsequent visit, the number of male flowers, female flowers, developing fruits (< 5 mm diameter) and mature fruits (> 5 mm diameter) were counted on each branch (Fig. 1). In total we conducted 70 surveys of flowering phenology across the four sites: 34 at Richmond and 12 at each of the other three sites. We assessed a total of 100,038 female flowers, 32,473 male flowers, 13,889 developing flowers and 12,098 mature fruits.

**Table 1:** Sampling locations and Australian Bureau of Meteorology (BOM) weather stations used for data analysis

	Grid reference	BOM Weather Station	Distance to station (km)
Millfield (MF)	− 32.8996, 151.2467	Pokolbin (061327)	6.2
Richmond (RC)	− 33.6194, 150.7378	Richmond (067021)	1.8
Shoal Bay (SB)	− 32.7176, 152.1673	Nelson Bay (061054)	1.3
Shellharbour (SH)	− 34.5945, 150.8984	Albion Park (068241)	3.1

All statistical analyses were conducted in R Studio (v1.0.153) [43], using R (V. 1.1.414) [44]. Significance was set at α < 0.05. We obtained all temperature and rainfall data from the Australian Bureau of Meteorology (Bureau of Meteorology, 2018) using the closest available weather station (< 7 km) at each sampling location (Table 1) to test for associations with flowering and fruiting.. Environmental variables included the sum of precipitation in the previous 14, 15–28 and 28–42 days and the sum of all rainfall across 42 days prior to each phenology observation. In addition, we calculated the average daily minimum and maximum temperatures over the 14 days prior to each phenology observation. Photoperiod is an important factor for the phenology of many Australian plants [4, 5, 46]. This was therefore included in our analysis as the duration of daylight hours on the day of observation, to the nearest minute, which we obtained from www.timeanddate.com for Sydney, NSW.

We used negative binomial generalised linear mixed models (GLMM) to determine the degree to which environmental (climate, daylength) variables influence flowering phenology, whilst controlling for the effects of site and plant. Negative binomial models are useful for modelling count data, such as ours, where a large proportion of “true” zero values results in high variance and over-dispersion [47]. We constructed separate negative binomial GLMMs for the number of male, female, developing and mature fruits using the "glmer.nb" function in Lme4 library (v1.1–15) [48], specifying a random intercept and slope for each site and plant. To determine the most parsimonious combinations of environmental variables on the number of male flowers, female flowers, developing fruits and mature fruits we used the "dredge" function in the MuMIn library [49] to perform model selection. For each reproductive structure (i.e., fruits and flowers), we created a negative binomial model containing all the independent environmental variables. Models were fitted using maximum likelihood. Where the most parsimonious model identified by dredge included environmental variables that were likely to be highly correlated (i.e., maximum, and minimum average daily temperature), we created separate models for each variable and compared them using likelihood ratio tests [50, 51], preferring the model with the lowest AICc score. To check our assumption of over-dispersion we created an alternative GLMM using Poisson regression that does not have an extra parameter for modelling over-dispersion and compared the two models using a likelihood ratio test. For each reproductive structure, negative binomial regression models performed significantly better than poison regression models (p < 0.0001) supporting our assumption of over-dispersion in the data.

### Pollinator phenology

We conducted surveys of *Epicephala* moths between one and four times per week at the Richmond field site only from 13/11/2017 to 01/04/2018. Multiple surveys per week were conducted during periods of high *Epicephala* activity (> 4 individuals observed on the first night of observation), otherwise surveys occurred once per week. *Epicephala* moth activity was observed shortly after sunset when the moths became active. Surveys were conducted by continuously walking along two perpendicular boardwalks forming an “X” shaped transect approximately 100 m in total length within the woodland. During surveys we counted the number of *Epicephala* moths observed on all plants in the transect over a period of 1 h using a white LED head torch*.* Although white lights were found to disturb pollination behaviours [35], using them gave a larger field of view and better chances of detecting *Epicephala* than red lights. No attempt was made to determine which *Epicephala* species were present, as this requires destructive abdominal dissections [35].

We used negative binomial regression to model the abundance of *Epicephala* moth species at the Richmond site over time. To do this, we calculated the mean number of *Epicephala* observed per night for each week by averaging counts of *Epicephala* across all the observations for each week (1–4 observations) from 13/11/2017 to 01/04/2018. For each week, we estimated the sum of male flowers, female flowers, developing fruits and mature fruits at the time of *Epicephala* moth observations using our phenological data. Pearson’s correlation coefficient tests showed that the mean abundance of *Epicephala* moths was significantly correlated with the sum of the number of male flowers, female flowers, developing fruits and mature fruits at the time of each pollinator observation (all |p|< 0.05). As such, we initially modelled *Epicephala* abundance as a function of the sum of male flowers, female flowers, developing and mature fruits using negative binomial regression in MASS package [52]. Although all four phenological variables correlated with *Epicephala* abundance, only the number of mature fruits was found to have a significant relationship (p < 0.05) with the mean number of pollinators observed. The sum of male flowers, female flowers and developing fruits was thereafter excluded from our model.

We then extended our model of *Epicephala* abundance by adding the variables that drive fruit production, specifically rainfall, temperature, and photoperiod. To do this we constructed a range of environmental variables to include in our extended model for each weekly average count of *Epicephala* moths. For rainfall we calculated the total rainfall (mm) in cumulative weekly intervals (0–7, 7–14, 14–21, 21–28 and 0–28 days) prior to each week of observations. For temperature, we calculated the sum of the minimum and maximum daily temperatures, over the same intervals as rainfall, prior to each week of observations. As photoperiod was found to be a significant predictor of the number of female flowers and developing and mature fruits, we also looked for associations between photoperiod and *Epicephala* abundance. To determine the most appropriate timescale for the environmental variables (i.e., 0–7, 7–14 days) we again used the "dredge" function in the MuMIn library [49] to perform model selection. We chose the model with the lowest AICc run for inclusion in our final model. The log likelihood of the alternative Poisson regression model was significantly lower than the negative binomial regression model (χ^2^ = 392.80, p = 0.007), again supporting our assumption of over-dispersion in the moth abundance data.

### Winter flower surveys

The results of our phenology surveys showed that female flowers were retained over the winter period and began developing into fruits before the appearance of male flowers or moth pollinators (Fig. 3). We therefore assessed whether the female flowers present on *B. oblongifolia* over the winter period had previously been pollinated. In the late winter of 2017, we collected ten female flowers from each of 15 *B. oblongifolia* bushes at Richmond. Flowers were selected haphazardly from separate branches on each plant. Flowers were taken back to the laboratory and dissected under a Leica EZ4 Stereo Microscope (Leica Microsystems, Wetzlar, Germany) to determine the number of pollen grains per flower and if any insects were present in the flowers. We used a Welch’s t-test to determine if there was a difference in the number of pollen grains in flowers with and without larval feeding damage. We tested for a difference among plants in the number of pollen grains per flower using a one-way ANOVA using the R stats package.

### Pre-pupal diapause

The results of our winter flower surveys suggested that *Epicephala* likely diapause as small eggs or young larvae in female *B. oblongifolia* flowers. However, we were also interested in determining if *Epicephala* moths may also diapause between the final larval instar and adult life history stages, as seen in *Yucca* moths. We collected 263 fruits from two successive crops at the Richmond site in the spring (November) (n = 115) and autumn of 2019 (April) (n = 148). One to ten fruits were collected haphazardly from ~ 15 adult *B. oblongifolia* plants. Fruits were placed individually in plastic emergence pots [36]. The pots were left outside in a shaded position to mimic ambient environmental conditions and checked at 1–2 week intervals. The type of insect, date of larval emergence and date of adult eclosion were recorded for all insects that emerged from the collected fruits. Where no insects emerged within ten weeks of collection, fruits were dissected and checked to see if they had previously contained insects. Collected *Epicephala* moths were identified by genital dissection [35].

## Results

### Flowering and fruiting phenology

**Fig. 2:**
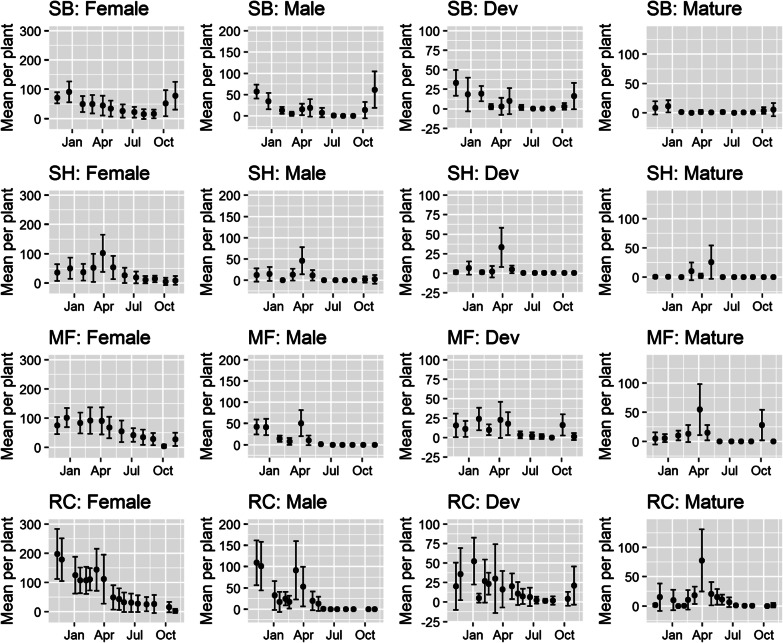
*Breynia oblongifolia* phenology by site. Mean number of male flowers, female flowers, developing fruits (Dev) and mature fruits per plant at Shoal Bay (SB), Shellharbour (SH), Millfield (MF) and Richmond (RC). Error bars show the standard deviation of the mean

Female flowers were generally present throughout the year, peaking in abundance during the spring or late summer and declining during winter to low but stable levels (Fig. 2). Interestingly, female flowers were retained on the plants even under periods of drought. For example, in the late winter and early spring of 2017 (July–September) *Breynia oblongifolia* plants at the Richmond site experienced a prolonged spell of unusually dry weather (Additional file 1). During this period, the whole population showed signs of water stress including leaf rolling and leaf abscission. However, many of the plants retained a large proportion of their female flowers, even when they were heavily defoliated. Both the sum of precipitation over the past 42 days (p < 0.0001) and photoperiod (p < 0.0001) were significant predictors of the number of female flowers in our best performing model (Table 2).

**Table 2:** GLMM coefficients for relationships between environmental variables and flowering and fruiting phenology in *Breynia oblongifolia* at the Richmond, Shellharbour, Shoal Bay and Millfield sampling sites.

	*β*	*SE*	*z*	*p*
Female flowers				
Intercept	3.7	0.182	20.47	< 0.0001
Rainfall^a^	0.23	0.029	7.97	< 0.0001
Daylight hours^b^	0.58	0.027	21.22	< 0.0001
Male Flowers				
Intercept	2.06	0.242	8.52	< 0.0001
Rainfall^a^	0.70	0.071	9.88	< 0.0001
Minimum Temp^c^	1.27	0.061	20.58	< 0.0001
Developing Fruits				
Intercept	1.65	0.33	4.88	< 0.0001
Rainfall^a^	0.50	0.050	10.08	< 0.0001
Daylight hours^b^	0.60	0.045	13.41	< 0.0001
Mature fruits				
Intercept	1.73	0.289	5.99	< 0.0001
Rainfall^a^	0.65	0.08	7.33	< 0.0001
Daylight hours^b^	0.16	0.067	2.46	0.0136

^a^Sum of precipitation over the past 42 days

^b^Photoperiod day on the day of observation

^c^Sum of daily minimum temperature over the 14–21 days prior to each phenology observation

**Fig. 3:**
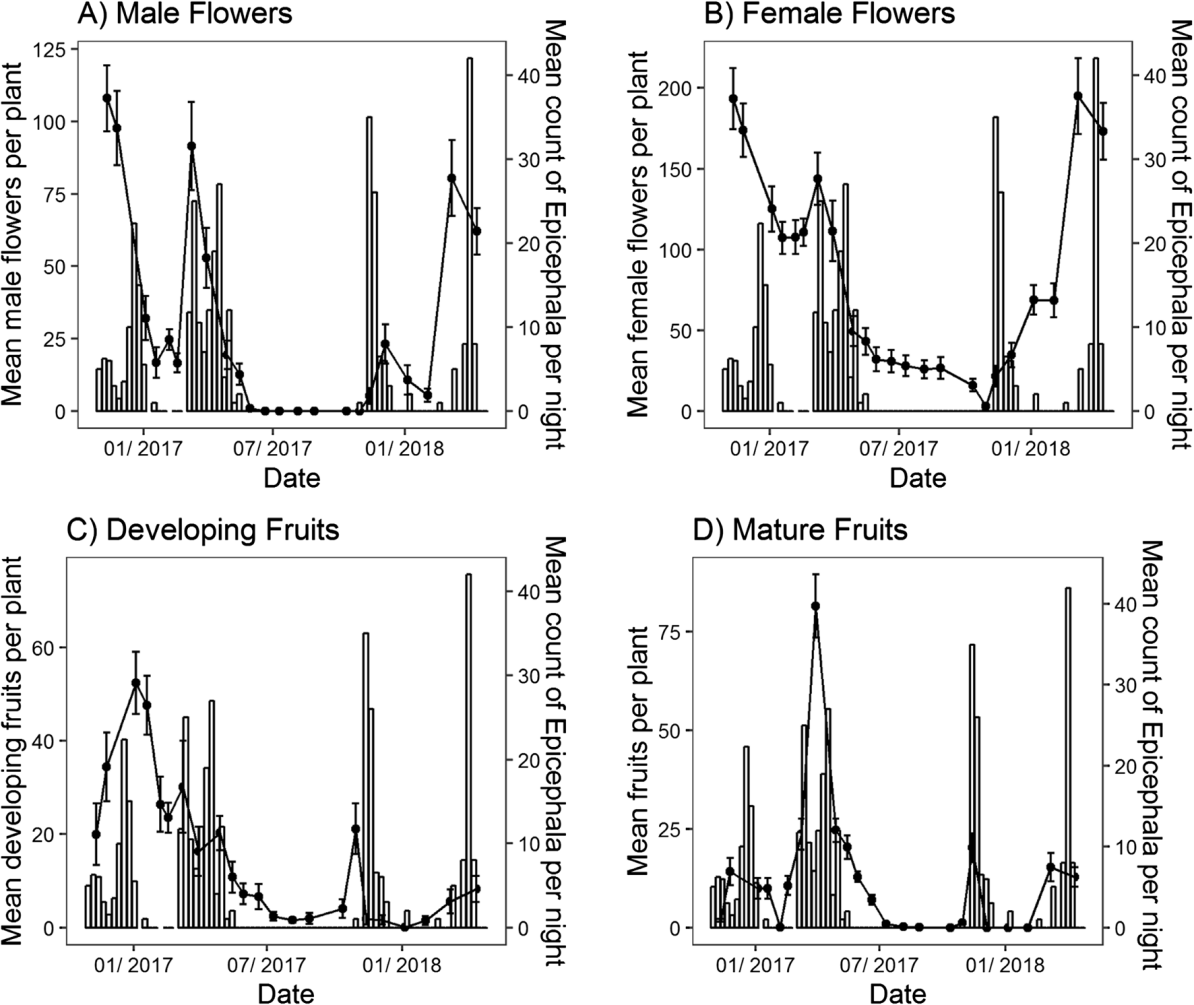
Mean weekly counts of *Epicephala* per night (bars) alongside the mean number of **A** male flowers, **B** female flowers, **C** developing fruits, **D** mature fruits per *Breynia oblongifolia* plant (lines ± SEM) at the Richmond site

Male flowers were most abundant during the spring and summer but declined rapidly in periods of particularly hot and dry weather (Jan-Feb) and were entirely absent during the winter months (May–August) (Figs. 2, 3). As with female flowers, the sum of precipitation over the past 42 days was a strong predictor of the number of male flowers (p < 0.0001). However, in contrast to female flowers, the average minimum temperature (p < 0.0001) was the best predictor of the number of male flowers across the four sites (Table 3).

**Table 3:** Coefficients for relationships between the number of mature fruits and temperature on *Epicephala* moth activity at the Richmond site

	*β*	*SE*	*z*	*p*
Intercept	− 1.3870	0.6177	− 2.245	0.02475
Total mature fruits^a^	0.0013	0.0004	3.231	0.00123
Minimum daily temp^b^	0.01962	0.0064	3.067	0.00216

^a^Total mature fruits at the time of observation

^b^The sum of minimum daily temperatures 14–21 days prior to each observation

*Breynia oblongifolia* plants usually produced 1–2 fruit crops per year and each crop lasted for 1–3 months. When combining across all sites, developing and mature fruits were present on plants throughout most of the year (July to May: Fig. 2) but there was considerable variation in fruit production among sites, even at the same time of year (Fig. 2). This is most likely because of differences in rainfall that varied among the four sites (Additional file 1). Fruit production did not usually occur during the hottest (Jan–Feb) and coldest parts of year (June and July), although there was some fruiting recorded in early July (mid-winter) at the Richmond site (Fig. 3). Interestingly, at this time *Epicephala* moths were not active (Fig. 3) and male flowers were also absent. Like female flowers, the number of developing fruits was best predicted by the sum of precipitation over the past 42 days (p < 0.0001) and photoperiod (p < 0.0001) (Table 2). Similarly, the abundance of mature fruits was best predicted by the sum of the precipitation over the past 42 days (p < 0.0001) and photoperiod (p = 0.0136) (Table 2).

### Pollinator phenology

*Epicephala* moth activity at the Richmond site varied widely over our 75-week observation period. Adults were active at the site for periods of 1–2 months in spring-early summer and then again in late summer-autumn period but were absent or undetected during the winter months (Fig. 3). In both 2017 and 2018, *Epicephala* were scarcely recorded for up to 3 months during mid-summer but quickly became very abundant again in late summer, with up to 42 individuals being recorded in a single hour-long observation period. Both the total number of mature fruits (p < 0.01) and the sum of minimum daily temperatures 14–21 days prior to each observation (p < 0.01) were significantly related to the abundance of *Epicephala* adults (Table 3). Rainfall was, however, not related to moth abundance (p > 0.05).

### Winter flower surveys

**Fig. 4:**
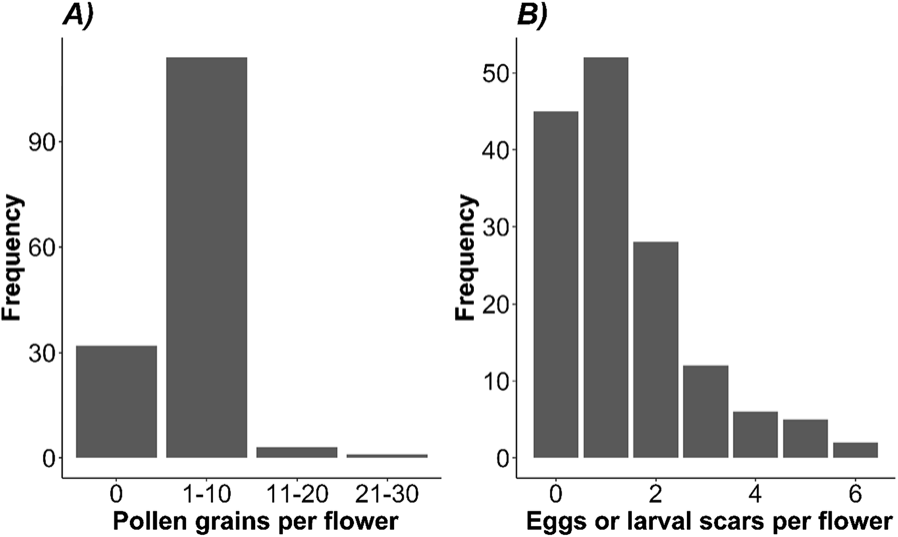
Frequency distributions of **A** pollen grains and **B** eggs or larval scaring in the overwintering female flowers of *Breynia oblongifolia* collected during in winter at the Richmond site (July 2017) (n = 150)

Many overwintering female flowers contained pollen and evidence of overwintering pollinator eggs or small larvae (Fig. 4). Of 150 inspected flowers, 118 had pollen grains on the surface of the stigma with an average of 10.9 (SD = 5.68) grains per flower. There was no significant difference among plants in the number of pollen grains per flower (*F*_1,148_ = 3.75, *p* > 0.05). Of the 150 flowers collected, 86 showed scars in the tissue of the ovary wall consistent with boring damage by *Epicephala* [36] and 47 contained at least one *Epicephala* egg or egg case (Fig. 4). These scarred flowers had between 1 and 4 scars (mean = 1.54, SD = 0.78) visible per flower. It was not possible to determine if these marks were attributable to multiple individuals or multiple boring attempts by the same individual. There was no difference in the number of pollen grains present in flowers with or without larval scarring (t = − 0.64, df = 130.9, p < 0.520).

### Pre-pupal diapause

**Fig. 5:**
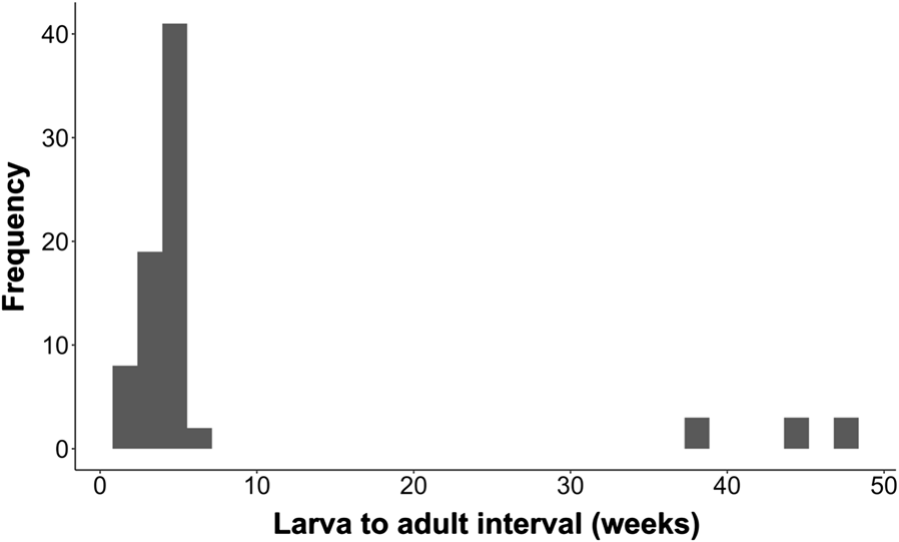
Frequency distribution of the number of weeks between the emergence of *Epicephala* larvae and their eclosion to adults (n = 78) from *Breynia oblongifolia* fruits collected at the Richmond site.

Of the 263 collected fruits, 129 contained no insects, 52 contained evidence of feeding damage but no insects (i.e., insects emerged before fruit collection), 4 contained non-*Epicephala* insects and 78 contained *Epicephala* larvae. Of the 78 *Epicephala* larvae that emerged from fruits in the spring and autumn crops, 69 (88%) pupated and eclosed as adults within 5 weeks with a mean time of emergence to adult eclosion of 3.6 weeks. Nine *Epicephala* (9/78) remained as larvae for between 37 and 48 weeks, successfully pupating and eclosing to adults from 9 months to nearly one full year after they emerged from fruits (Fig. 5). All long diapausing moths were from the autumn crop. Five were male *Epicephala* sp. B and the remaining four were an equal proportion of male and female *Epicephala* sp. A. Only 13 *Epicephala* were collected from the spring crop, all of which pupated within 5 weeks of emergence.

## Discussion

**Fig. 6:**
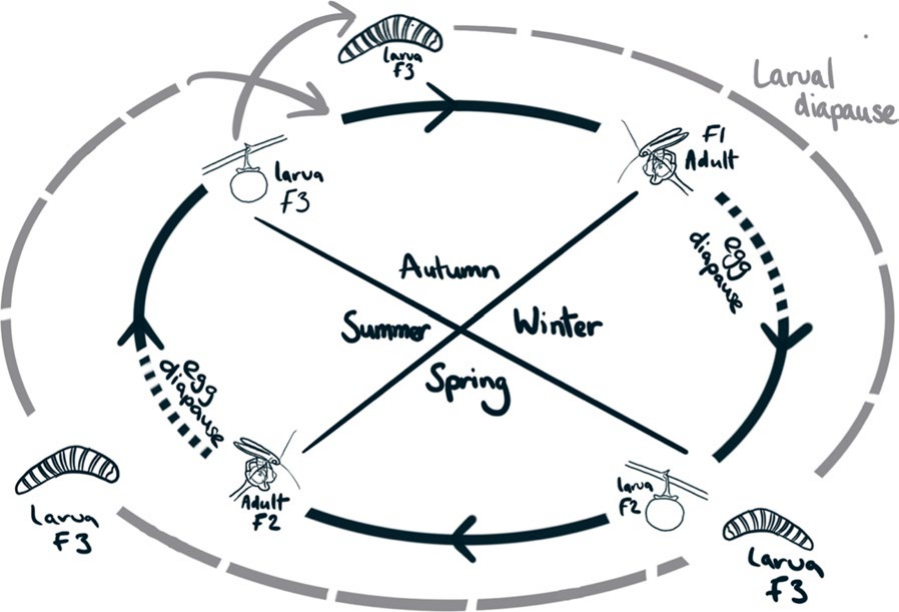
Diagram of the proposed lifecycle of *Epicephala* moths on *Breynia oblongifolia* over three successive generations (F1–3). The black cycle denotes the life history for the majority of individuals, in which moths go through two generations per year with the winter months spent as eggs or small larvae in overwintering female flowers. Egg diapause may also occur during the summer months in periods with insufficient rainfall to initiate fruiting. The grey cycle denotes the alternative strategy taken by 12% of individuals in the autumn crops, in which pre-pupal larvae emerge from fruits and enter diapause for 38–48 weeks, re-joining the general population 1–2 generations later. The number of generations skipped depends on the duration of the diapause. Here, a pre-pupal diapause of 52 weeks is depicted

*Epicephala* moths appear to use diapause at multiple stages in their lifecycle in order to deal with variable flowering phenology (Fig. 6). The close association between the abundances of fruits and moths suggested that moths may diapause as eggs or young larvae within pollinated flowers, appearing as adults as fruits mature. We confirmed this by showing that many overwintering flowers contained pollen and evidence of diapausing pollinators. Our observations suggest egg diapause within dormant flowers serves a critical function of physically linking the pollinator lifecycle to host plant flowering phenology, ensuring that pollinator emergence coincides with flowering. In addition, our study of adult eclosion times also indicate that 12% of the autumn generation of *Epicephala* enter a pre-pupal diapause, emerging up to one year later. As such, *Epicephala* moths appear to use diapause at multiple stages in their lifecycle in order to track host plant phenology and mitigate against environmental unpredictability.

### Host plant phenology

Inter-annual rainfall can be highly variable within Australia, even when compared to environmentally similar areas elsewhere in the world [37, 53]. Indeed, many species of Australian plant are known to fruit and flower in response to variation in both rainfall and photoperiod [4, 5]. In *B. oblongifolia*, female flowers were present all year round, but their abundance varied widely and was best predicted by rainfall and photoperiod. In this way, *B. oblongifolia* seems typical of many Australian plants in that its reproductive phenology is adapted to environmental unpredictability. The effect of rainfall on a plant’s phenology is likely to be dependent on characteristics of the soil and surrounding landscape, as well as the frequency and intensity of previous rainfall events [6, 54, 55]. Given that soil moisture more accurately reflects the total water available to each individual plant, we believe that it is likely that soil moisture would also be a significant predictor of flowering and fruiting phenology if included in our model.

Interestingly, our winter flower surveys show that *B. oblongifolia* frequently retains pollinated flowers over winter. Our phenological model found that *B. oblongifolia* sets fruit in response to rainfall. Together, these two results suggest that *B. oblongifolia* can delay fruit maturation in pollinated flowers over the winter period and potentially also through the spring and summer depending on the prevailing environmental conditions. This likely explains why only female flowers are present during the winter, as well as how some fruits began to develop in the late winter of 2017, when both *Epicephala* moths and male flowers were absent (Fig. 3). Indeed, our own flower bagging experiments support this explanation, with many bagged (i.e., pollinator-excluded) branches developing fruits [56]. Retaining previously pollinated flowers that can develop to fruits in response to largely unpredictable rainfall is likely to make *B. oblongifolia* less vulnerable to environmental variability, thereby reducing the risk of fruits developing during periods of drought and potential reproductive failure.

The exact mechanism that allows *B. oblongifolia* to retain pollinated flowers and then develop them to fruits months later remains unclear. Pollen can remain viable for several hours to several months depending on the species and environmental conditions [57–60]. It may be the that the pollen on the overwintering flowers of *B. oblongifolia* have yet to germinate and does so shortly before fruit development. Currently, it is not known how long the pollen of *B. oblongifolia* can remain viable under natural environmental conditions. The fruits of several important crop plants are known to arrest their development in response to climatic conditions, with fruit growth initiating again following pollen fertilisation [61, 62]. As such, an alternative explanation could be that the retained pollinated flowers are already fertilised but remain in a developmental pause over winter until released by an environmental cue, such as lengthening photoperiod or increased soil moisture following rainfall. Further experimentation is required to answer these questions.

### Pollinator phenology

*Epicephala* abundance at the Richmond field site in both the spring and summer of 2016–2017 and 2017–2018 occurred in two discrete peaks of high abundance, corresponding with similar peaks in fruit abundance (Fig. 3). Our analysis showed that the number of mature fruits was a significant predictor of *Epicephala* abundance. This is most likely because *Epicephala* moths emerge from mature fruits as larvae and then pupate to adults [14, 20]. The appearance of fruits is therefore an important predictor of adult moth abundance, as observed in some other leaf flower species [19–21]. Although we found no statistical evidence for a relationship between rainfall and moth abundance, there is an indirect relationship between the abundance of moths and rainfall via the abundance of fruits, which is rainfall dependent. Thus, rainfall and photoperiod are critical factors in the phenology of the *B. oblongifolia* host plant and, by extension, its *Epicephala* pollinators.

Consistent with our prediction, the abundance of both male flowers and *Epicephala* moths were best predicted by the mean minimum temperature 14–21 days prior to each plant and pollinator survey. Lepidoptera are often phenologically synchronised with their host plant’s growth stages through temperature [30–34]. As male flowers are a source of essential pollen and potentially nectar [19, 20], female *Epicephala* moths are likely to be under selection to synchronise their adult eclosion with the occurrence of male flowers. The fact that both male flowers and moths respond to the mean minimum temperature three weeks prior to each observation probably reflects the developmental time between the environmental trigger and appearance of mature fruits and moths. Indeed, the average time between the emergence of larvae from fruits and adult eclosion in our eclosion study was 3 to 4 weeks, strongly suggesting that temperature plays a critical role in synchronising moth and pollinator life histories.

The absence of adult moths during the winter months (June–August) and hottest summer months indicates that moths probably diapause during this stage in their lifecycle (Fig. 4). Furthermore, the close association between fruits and the abundance of adult moths suggests that these insects diapause as eggs, or possibly young larvae, within flowers that then develop to fruit. In line with our expectations, when we examined overwintering flowers, many showed scarring and boring damage consistent with *Epicephala* moths and their larvae [35, 36]. Although we did not survey flowers during the hottest summer months, we believe that it is very likely that moths also diapause as eggs in female flowers during this time. This is because all spring emerging larvae pupated directly to adulthood and did not enter a pre-pupal diapause. As such, spring emerging *Epicephala* presumably eclose, mate and lay diapausing eggs in female flowers that are present through the summer when adults are absent. Periods of egg dormancy are known in other *Epicephala* species [21] and many Australian Lepidoptera are also known to exhibit extended periods of egg diapause [27–29]. The larvae of some fig wasps are also known to overwinter within dormant fig flowers in temperate climates [22]. Our analysis of the phenological data has demonstrated that in *B. oblongifolia*, fruiting and flowering are phenologically synchronised with both fruits and new flowers appearing together. As such, egg or young larval diapause likely ensures that overwintering larvae develop and emerge at, or near, the time of flowering, creating a physical link between plant and pollinator lifecycles.

In moths, diapause can occur at both the egg and pre-pupal life history stages. Our study of the interval between larval emergence and eclosion found that 12% of the *Epicephala* larvae that emerged in the autumn entered a pre-pupal diapause for periods of up to 48 weeks. These long diapausing *Epicephala* included both species known to pollinate *B. oblongifolia* [35]. The majority of moths that emerged in the autumn pupated directly to adulthood, eclosing 3 to 4 weeks after leaving the fruits. Spring emerging *Epicephala* were not observed to enter an extended pre-pupal diapause, but this may reflect the lower sample size (n = 13). The varying lengths of pre-pupal diapause documented here may constitute a bet-hedging or risk-spreading strategy [63]. Bet-hedging strategies involve a loss of individual fitness to reduce variance in fitness over time, thereby increasing the long term (geometric) mean fitness of the genotype or lineage [64, 65]. Such strategies are adaptive in unpredictable environments, where variance in fitness between generations is high. In our example, moths that pupate directly to adults after emerging from fruits late in the growing season may experience shortages of flowers. A bet-hedging genotype that produces multiple phenotypes (e.g., differing diapause durations) may thereby achieve greater geometric fitness over time by reducing variance in fitness between flowering seasons. Bet-hedging strategies are commonly inferred in insects, but rarely shown definitively [63]. This is because such strategies must be correctly distinguished from genotypic polymorphism within populations [63]. Furthermore, demonstrating that bet-hedging strategies occur requires demonstrating greater geometric fitness in bet-hedging genotypes compared to none bet-hedging genotypes. Alternatively, it is also possible to demonstrate bet-hedging by showing variation in the trait in question (e.g., diapause duration) in relation to environmental uncertainty. Bet-hedging is, therefore, challenging to demonstrate experimentally. Regardless, our work is the first to suggest the possibility of a bet-hedging strategy in a nursery pollination mutualism.

### Implications for the mutualism

Since they represent independent evolutionary origins of pollination mutualism, it is interesting to compare the phenology of plants and pollinators in the fig, *Yucca,* and leaf flower mutualisms*.* Based on available data*,* it would seem that, at the population level, the flowers of both fig trees and leaf flowers are generally present throughout the year [18–21, 24, 25]. However, as this study and others have demonstrated [21], flowers may be present but dormant for part of that time. In at least one temperate fig species, overwintering flowers act as refuges for fig wasp pollinators through the winter months [22]. In *B. oblongifolia*, and possibly other leaf flower plants, the majority of plants bear female flowers year-round, with low numbers also present during the winter. Year round flowering may thereby promote stable populations of *Epicephala* moths by providing a refuge for pollinators during periods when plants are not growing fruits or new flowers. This could explain why some *B. oblongifolia* plants maintained female flowers during periods of obvious drought stress in the unusually dry winter of 2017. Indeed, our observation that these pollinated and pollinator-containing flowers were maintained at the expense of leaf tissues suggests a high fitness value to the host plants. In the *Yucca*-yucca moth OPM, pollinators diapause as pre-pupal larvae in the soil around their host plants and may wait several years between the appearance of flowers [11]. The ability of yucca moths to diapause for multiple years between flowering events probably also promotes the stability of pollinator populations. Our study has found that both year-round flower provision and pre-pupal diapause occur in at least some leaf flower plants, showing similarities with both the *Yucca* and fig mutualisms.

Egg diapause in *Epicephala* may have other important implications for the mutualism. Overwintering larvae or eggs are likely to suffer moderate levels of mortality during diapause [66, 67]. This increased mortality may benefit the plant by reducing the number of seeds that are consumed by pollinators. Overwintering mortality could help to explain the large proportion of *B. oblongifolia* fruits (10–30%) that do not contain *Epicephala* larvae [36]. Fruits that do not contain pollinators are generally more frequent in crops collected in the spring and contribute a high proportion of the intact seeds produced across *B. oblongifolia* populations [36]. As such, egg diapause mortality may be an important factor in reducing seed destruction by pollinating seed herbivores, thereby helping to maintain the mutualism in the face of competing interests between mutualists.

## Conclusions

*Epicephala* moths appear to use diapause at multiple stages in their lifecycle in order to survive through periods of variable plant phenology. Furthermore, moth abundance is predicted by the same environmental variables as male flowers, suggesting that moths track flowering through temperature. These adaptations may thereby mitigate against unpredictability in the timing of fruiting and flowering because of variable rainfall. This is the first study of how *Epicephala* moths track host plant phenology and avoid potentially disastrous phenological mismatches. How widespread egg and pre-pupal diapause are within *Epicephala* moths remains to be seen. Such traits may have important implications for both the evolutionary stability of these mutualisms and their rapid diversification.

## Supplementary Information

Additional file1 (PNG 215 KB)

## Data Availability

The datasets supporting the conclusions of this article have been made publicly and permanently available in the Figshare online repository under the following DOIs: winter flower surveys https://doi.org/10.6084/m9.figshare.14623872.v1, flowering and fruit phenology, https://doi.org/10.6084/m9.figshare.14623641.v3, pollinator phenology, https://doi.org/10.6084/m9.figshare.14623563.v3,and pre-pupal diapause https://doi.org/10.6084/m9.figshare.14625531.v1.
