# Supplementary figures and images for: Staying in touch: how highly specialised moth pollinators track host plant phenology in unpredictable climates
Source: BMC Ecol Evol. 2021 Aug 24;21:161. doi: 10.1186/s12862-021-01889-4 (PMC8383429; doi:10.1186/s12862-021-01889-4)

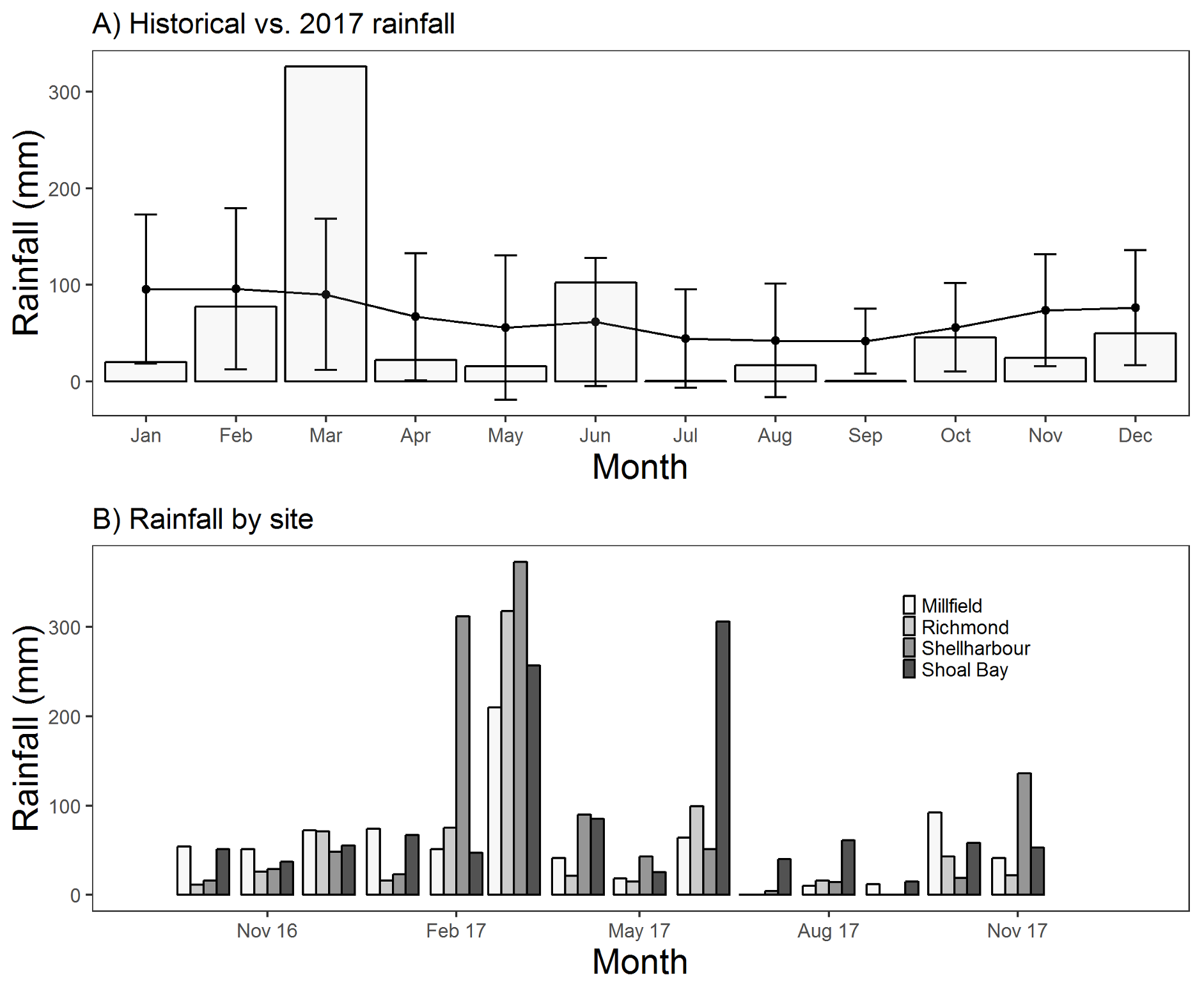

Supplement: Supplementary file 1 — Additional file1 (PNG 215 KB) [file 12862_2021_1889_MOESM1_ESM.png]
